# Antioxioxidant and antiapoptotic effects of Thymosin β4 in Aβ-induced SH-SY5Y cells via the 5-HTR1A/ERK axis

**DOI:** 10.1371/journal.pone.0287817

**Published:** 2023-10-03

**Authors:** Gui-Hong Zhang, Kai Ling Chin, Shi-Yan Yan, Rahmawati Pare

**Affiliations:** 1 School of Medicine, Xi’an International University, Xi’an, Shaanxi, China; 2 Faculty of Medicine and Health Sciences, Department of Biomedical Science, Universiti Malaysia Sabah, Kota Kinabalu, Sabah, Malaysia; 3 International Innovation Institute of Acupuncture and Moxibustion, Beijing University of Chinese Medicine, Beijing, Hebei, China; University of Nebraska-Lincoln, UNITED STATES

## Abstract

Alzheimer’s disease (AD) is a common amnestic cognitive impairment characterised by β-amyloid (Aβ) plaques deposit in the brain of the elderly. AD is a yet incurable disease due to its unknown exact pathogenesis and unavailability of effective remedies in clinical application. Thymosin β4 (Tβ4) is a housekeeping protein that plays important role in cell proliferation, migration and differentiation. It has the ability to protect and repair neurons however it is still unclear involvement in AD. Therefore, the aim of this study is to elucidate the role and mechanism of Tβ4 in mediating the improvement of AD. AD-like cell model was constructed in neuroblastoma cell line SH-SY5Y treated with Aβ. Overexpression of Tβ4 were done using lentivirus infection and downregulation through siRNA transfection. We performed western blot and flow cytometry to study the apoptosis and standard kits to measure the oxidative stress-associated biomarkers. There is significant increased in viability and decreased apoptosis in Tβ4 overexpression group compared to control. Furthermore, overexpression of Tβ4 suppressed the expression of pro-apoptotic markers such as Caspase-3, Caspase-8, and Bax meanwhile upregulated the expression of anti-apoptotic gene Bcl-2. Tβ4 alleviated oxidative damage by reducing MDA, LDH and ROS and increasing SOD and GSH-PX in Aβ-treated SH-SY5Y cells. We found that Tβ4 inhibit ERK/p38 MAPK pathway and intensify the expression of 5-HTR1A. Additionally, we showed that upregulation of 5-HTR1A dampened the Tβ4 to activate ERK signalling. In conclusion, our study revealed the neuroprotective role of Tβ4 in AD which may open up new therapeutic applications in AD treatment.

## Introduction

AD is the most common neurodegenerative disorder seriously endangering the physical and mental health of the elderly, with progressive cognitive dysfunction, memory loss and mental behavior symptoms (such as anxiety, depression, and delusions) as the main clinical manifestations [1–3]. Although the pathogenesis of the disease is not clear, there are some common characteristics like extracellular senile plaques deposition formed by the aggregation of Aβ in the cerebral cortex and hippocampus, and intraneuronal fibrillary tangles formed by the abnormal aggregation of Tau protein in the patients’ brains [4–6]. Although novel therapies such as music therapy [7] or combined therapy [8] were developed, there are still no effective treatments and drugs in clinical practice.

Mitogen-activated protein kinases (MAPK) are a group of important signal transmitters from the cell surface to the nucleus. They play vital roles in cell biological reactions such as cell proliferation, differentiation, transformation, and apoptosis [9]. Studies have shown that the MAPK signaling pathway also exerts a vital function in the regulation of anxiety and depression [10,11]. For example, depression and anxiety-like behaviors induced by chronic social defeat stress may be ameliorated by inhibiting the p38-MAPK signaling pathway in mouse hippocampal neurons [12,13]. These findings indicate that regulating the MAPK signal transduction pathway may also improve anxiety and depression in AD patients.

Tβ4 is a multifunctional polypeptide widely distributed in mammals and other vertebrates and has attracted much attention [14]. Although its molecular mechanisms are not clear, Tβ4 is involved in many physiological and pathological processes, such as wound healing, tumor metastasis, angiogenesis, apoptosis, corneal and myocardial repair [15]. It is also involved in anti-inflammatory and neurodegenerative processes [16], especially in nervous system development and repair [17,18]. As a polypeptide that inhibits inflammation [19], improves nerve axon regeneration [20,21] and nerve development [22], and affects glial cell differentiation [23]. With the in-depth study of the function and regulating mechanism of Tβ4, new treatments of neurological diseases, especially a series of neurodegenerative diseases like AD may be found [24]. Nevertheless, it is still unclear whether Tβ4 can improve mental and behavioral disorders such as anxiety and depression symptoms or not.

In the current study, we aimed to study whether Tβ4 has neuroprotective effects on Aβ induced damage of human neuroblastoma SH-SY5Y cells and investigate the possible underlying mechanisms.

## Materials and methods

### Cell culture and treatment

Human neuroblastoma cell line SH-SY5Y was purchased from American Type Culture Collection (ATCC, Manassas, VA, USA) and cultured in Dulbecco’s modified Eagle’s medium (DMEM, Gibco, MA, USA) supplemented with 10% fetal bovine serum (Gibco, MA, USA) and 1% penicillin-streptomycin. Cells were cultured in a 5% CO_2_ incubator at 37°C. SH-SY5Y cells were treated with Aβ (C008-4, Meilunbio, Dalian, China) for 48 hours to establish the *in vitro* AD model.

### Vector construction, lentivirus package and siRNA transfection

Tβ4 overexpression vector was constructed by cloning TMSB4X ORF (NM_021109.4) into pCDH-CMV-MCS-EF1-GFP-Puro vector (MiaolingBio, Wuhan, China). Lentivirus was packaged using HEK293Lenti cells and the lentiviral packaging vector (System Biosciences, Palo Alto, CA, USA) following the manufacturer’s instruction. SiRNAs targeting 5-HTR1A were purchased from RiboBio (Guangzhou, China) and transfected into SH-SY5Y cells using lipofectamine 3000 (Invitrogen, USA).

### Cell viability

Cell viability was analyzed using Cell counting kit-8 (CCK-8, 7sea Biotech, Shanghai, China) assay. Briefly, SH-SY5Y cells were seeded into 96-well plate and treated with Aβ for 48 hours. Then 10 μL CCK-8 solution was added to each well and incubated for 2 hours at 37°C. Then the absorbance at 450 nm was measured using a microplate reader.

### Real-time quantitative polymerase chain reaction (RT-qPCR)

Total RNA was purified from SH-SY5Y cells using Trizol (Magen, China) and reverse-transcribed into cDNA using a HiFiScript cDNA synthesis kit (#CW2569M, CWbiotech, China). RT-qPCR was performed using SYBR Green qPCR Master mix (Vazyme, Q311-02, China). The primer sequences for TMSB4X and internal control GAPDH were listed as following: TMSB4X-Forward 5’-ACAAACCCGATATGGCTGAG-3’, TMSB4X-Reverse 5’-GAAGGCAATGCTTGTGGA-3’; GAPDH-Forward 5’-ATGGGGAAGGTGAAGGTCG-3’, GAPDH-Reverse 5’-GGGGTCATTGATGGCAACAATA-3’. Relative expression was calculated using the 2^-ΔΔCt^ methods.

### Western blot

Protein samples were prepared from treated SH-SY5Y cells using RIPA buffer (#WB053, HAT, China) and separated by SDS-PAGE and then transferred onto polyvinylidene fluoride (PVDF) membranes (#IPRH00010, Immobilon-P, China). After blocking with 5% non-fat milk, membranes were incubated with primary antibodies overnight at 4°C. Then membranes were washed with Tris-buffered saline containing Tween 20 and then incubated with horseradish peroxidase-conjugated secondary antibodies for 1 hour at room temperature. Protein expression was visualized using an enhanced chemiluminescence detection kit (#180–5001, Tanon, China). Antibodies used in the study were listed as below: anti-Tβ4 (19850-1-AP, Proteintech, China), anti-β-actin (20536-1-AP, Proteintech, China), anti-5-HTR1A (ab85615, Abcam, USA), antiHTR2C (ab124951, Abcam, USA), cleaved Caspase 3 (ab32042, Abcam, USA), cleaved Caspase 8 (9496, Cell Signaling Technology, USA), Bcl-2 (12789-1-AP, Proteintech, China), Bax (50599-2-Ig, Proteintech, China), p-ERK (ab201015, Abcam, USA), ERK (ab184699, Abcam, USA), pp38 (ab178867, Abcam, USA), p38 (ab170099, Abcam, USA), p-JNK (4668, Cell signaling Tech, USA), JNK (9258, Cell signaling Tech, USA), and anti-rabbit IgG (BL003A, Biosharp, China).

### Measurement of Malondialdehyde (MDA), Superoxide dismutase (SOD), Lactate dehydrogenase (LDH), and Glutathione peroxidase (GSH-PX) levels

The levels of MDA, SOD, LDH and GSH-PX in SH-SY5Y cells were analyzed according to the manufacturers’ instructions. MDA assay kit was obtained from Solarbio (#BC0025, Beijing, China). SOD activity was analyzed by using Total Superoxide Dismutase Assay kit with WST-8 (#S0101S, Beyotime, China). LDH assay kit was purchased from Nanjing Jiancheng Bioengineering Institute (#A020-2-2, Nanjing, China). GSH-PX was analyzed by using Cellular Glutathione Peroxidase Assay kit with NADPH (#S0056, Beyotime, China).

### Reactive oxygen species (ROS) production

Cellular ROS production was analyzed by using dihydroethidium (DHE, Cat# 50102ES02, Yeasen Biotech, China). Briefly, 5 μM DHE was added into the culture medium of SH-SY5Y cells and incubated at 37°C for 20 min. Then cells were washed with PBS, and fluorescence of DHE was detected using a confocal microscope or using flow cytometry (BD FACS Calibur, San Jose, CA, USA).

### Flow cytometry

SH-SY5Y cell apoptosis was detected using the Annexin V-FITC/PI apoptosis detection kit (BD Biosciences, USA). Cells were harvested, washed with PBS, and re-suspended in 500 μl binding buffer provided in the kit. Then, cells were incubated with 5 μl Annexin V-FITC and 1 μl PI at room temperature for 15 min in the dark. The percentage of apoptotic cells was detected by flow cytometry (Beckman Coulter Epics Xl, Beckman Coulter Life Sciences, Indianapolis, IN, USA).

### Statistical analysis

Statistical analysis was conducted using GraphPad Prism (V8.0, Graphpad Software, USA). The results were presented as means ± standard deviations (SD). Statistical differences between multiple groups were calculated by using the one-way ANOVA test followed by Tukey’s *post hoc* test for multiple comparions. A p < 0.05 was considered statistically significant.

## Results

Among various serotonin receptors, 5-HTR1A is closely related to AD [25], because they are highly expressed in the human hippocampus and are known to be involved in the regulation of memory processes. Studies have reported that compared with the control group, the density of 5-HTR1A in all cortical areas of AD patients such as frontotemporal dementia patients [26], hippocampus and raphe nucleus [27] decreased. A study has shown that inhibition of phosphorylation activation of ERK1/2 can reverse the decline of 5-HTR1A induced by Aβ [28]. Tβ4 has neuroprotective and repair effects, and may act as a nerve repair agent by promoting the differentiation and migration of neural stem cells to the damaged brain area [29]. However, whether Tβ4 can reduce Aβ-induced neurotoxicity through the potential connection of MAPKs (p38, ERK, JNK), 5-HTR1A and 5-HTR2C is still unclear.

### Tβ4 overexpression increases cell viability in SH-SY5Y cells against Aβ-induced cytotoxicity

**Fig 1:**
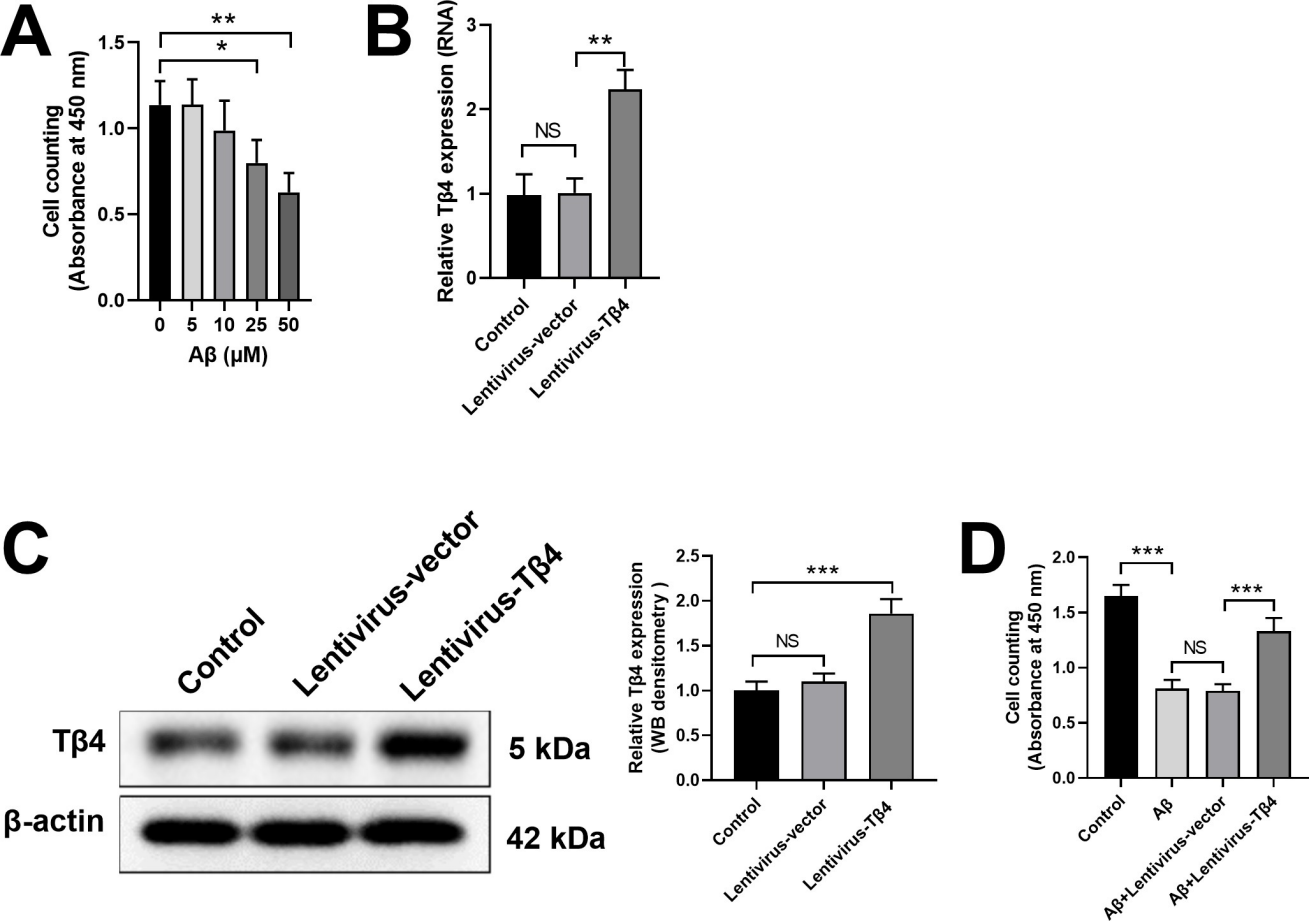
Neuroprotective effects of Tβ4 on Aβ-induced cytotoxicity in SH-SY5Y cells. (A) SH-SY5Y cells were treated with different doses of Aβ and the cell viability was analyzed. (B, C) SH-SY5Y cells were transduced with lentivirus empty vector or lentivirus vector overexpressing Tβ4. The relative Tβ4 mRNA expression (B) and protein expression (C) in SH-SY5Y cells were analyzed by qPCR and western blot 48 hours later. (D) SH-SY5Y cells were transduced with lentivirus empty vector or lentivirus vector overexpressing Tβ4, with or without 25 μM Aβ treatment for 48 h. The neuroprotective effect of Tβ4 on Aβ-induced cytotoxicity was analyzed by cell viability. Data are presented as mean ± SD (n = 3). NS, not significant; *, p < 0.05; **, p < 0.01; ***, p < 0.001.

To evaluate the cytotoxicity of Aβ in SH-SY5Y cells, we treated SH-SY5Y cells with various doses of Aβ and found the cell viability decreased in a dose-dependent manner (Fig 1A). We chose the minimum effective dose of Aβ (25 μM) for the subsequent experiments. To investigate the protective function of Tβ4 against Aβ treatment, we established a Tβ4-overexpression cell line using lentivirus vector transduction. As shown in Fig 1B, relative Tβ4 mRNA expression in Lentivirus-Tβ4-transduced SH-SY5Y cells was significantlyhigher than that in the Control or Lentivirus empty vector group. In addition, western blot result also confirmed that the protein level of Tβ4 remarkably increased in Lentivirus-Tβ4-transduced SH-SY5Y cells (Fig 1C). When treated with 25 μM Aβ for 48 h, the cell viability of Aβ+Lentivirus-Tβ4 group was significantly higher than empty vector group, indicating that Tβ4 had neuroprotective effects on Aβ-induced cytotoxicity (Fig 1D).

### Tβ4 overexpression alleviates oxidative stress levels in Aβ-treated SH-SY5Y cells

It has been reported that Aβ could elevate the oxidative stress level in cells by increasing ROS production [30]. Oxidative stress may occur when there is a disproportion between the production and removal of ROS [31,32]. ROS is a metabolic by-product in biological systems that has a double-edged role on cell function depending on its concentration. While low level of ROS can have a positive effect, excessive ROS can cause damage to cells [33,34].

**Fig 2:**
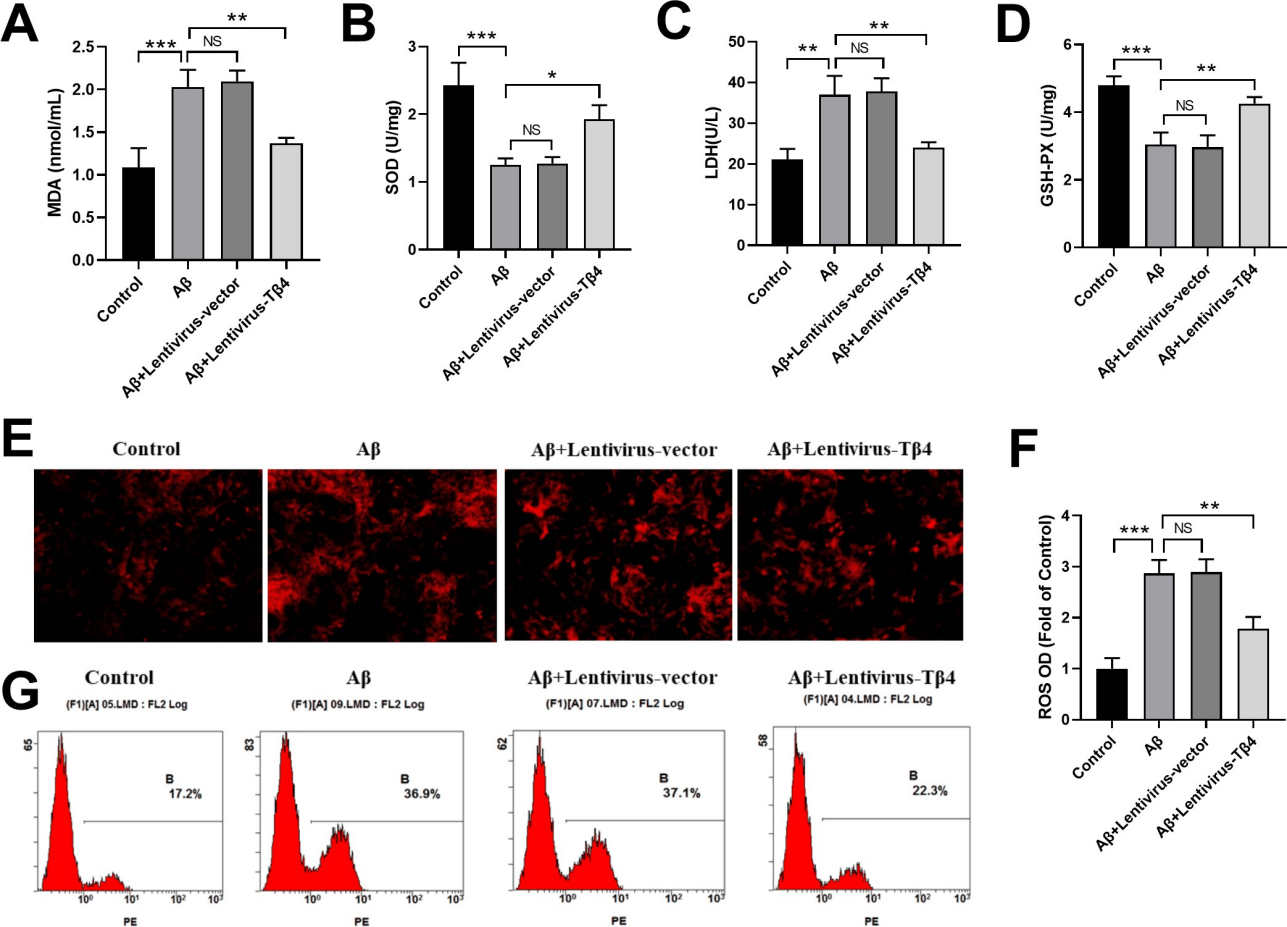
Effects of Tβ4 on MDA, SOD, LDH, GSH-PXand ROS in Aβ-treated SH-SY5Y cells. SH-SY5Y cells transduced with Lentivirus empty vector or Lentivirus-Tβ4 were treated with Aβ at the concentration of 25 μM Aβ, or left untreated for 48 hours. The levels of MDA (A), SOD (B), LDH (C), and GSH-PX (D) in SH-SY5Y cells were analyzed by using commercial kits. (E) Cells were stained with DHE, observed under a fluorescence microscope, detected by a fluorescence microplate reader and expressed as fold of non-treated normal group (F). (G) The fluorescence intensity in different cells was analyzed by flow cytometry. The data are shown as mean ± SD of three independent experiments each in triplicate. Scale bars, 100 μm. NS, not significant; *, p < 0.05; **, p < 0.01; ***, p < 0.001.

To investigate oxidative stress activity, we examined the tissue damage levels through MDA and LDH biomarkers and the protection against oxidative stress through antioxidant enzyme such as SOD and GSH-Px in Aβ-treated SH-SY5Y cells. As expected, Aβ treatment increased MDA and LDH levels, while decreased SOD and GSH-PX activities dramatically (Fig 2A–2D). Interestingly, overexpression of Tβ4 by lentivirus-Tβ4 transduction effectively reversed these effects, leading to enhancement in SOD and GSH-PX activities, and a reduction in LDH and MDA level (Fig 2A–2D).

To further evaluate the oxidative stress activity in Aβ-treated SH-SY5Y cells, we used DHE staining to label the ROS in cells. We found Aβ significantly increased ROS production, while Lentivirus-Tβ4 transfection inhibited this tendency (Fig 2E and 2F). The results were also confirmed by flow cytometry showing that the fluorescence intensity was higher in Aβ treatment group than that in the control group, while Lentivirus-Tβ4 transfection inhibited ROS production and decreased fluorescence intensity (Fig 2G).

### Tβ4 overexpression inhibits Aβ-induced apoptosis and proapoptotic biomarkers in SH-SY5Y cells

**Fig 3:**
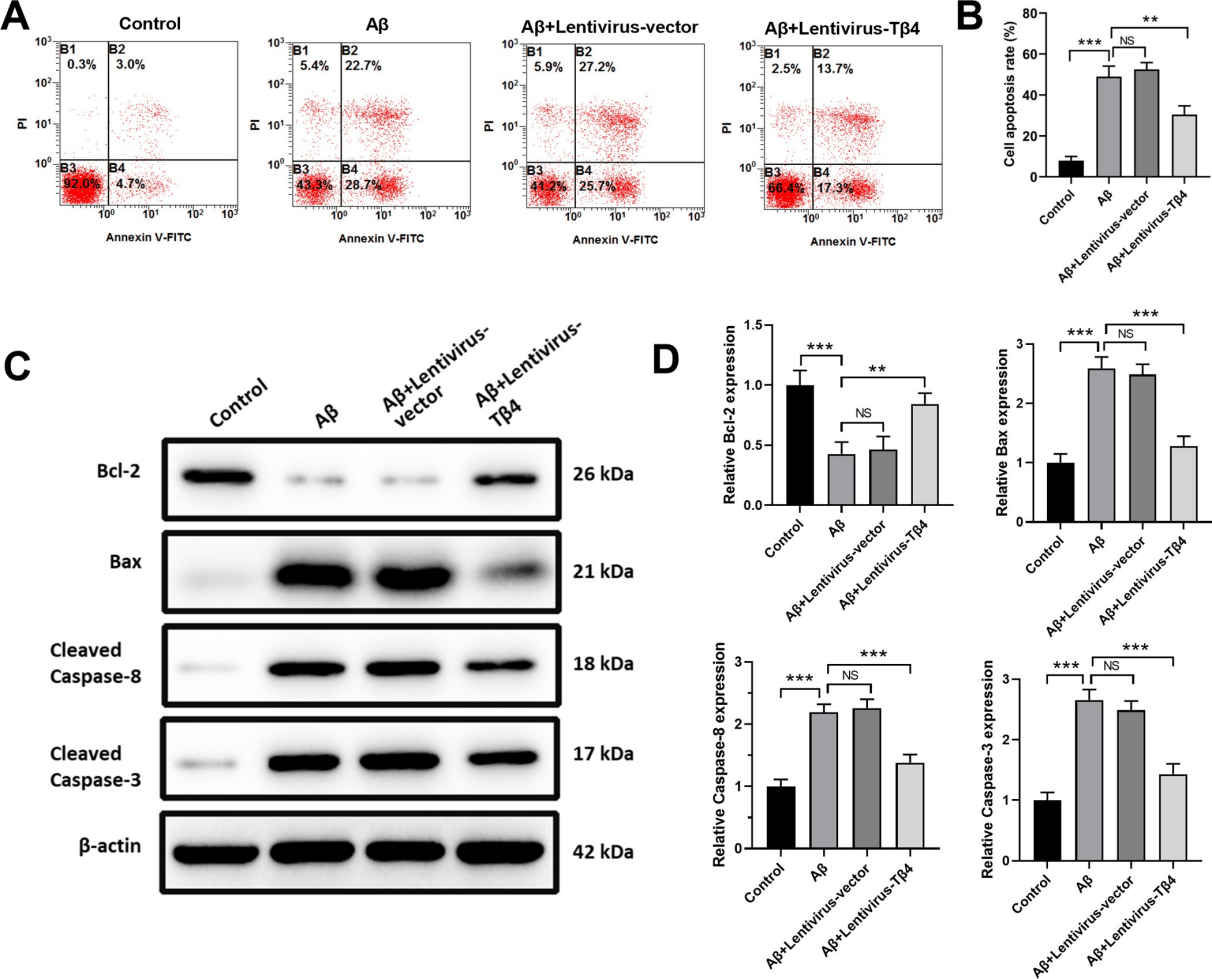
Effects of Tβ4 on Aβ-induced apoptosis and apoptosis-associated biomarkers in SH-SY5Y cells. SH-SY5Y cells with Lentivirus empty vector or Lentivirus-Tβ4 transduction were treated with 25 μM Aβ, or left untreated for 48 hours. Then cells were stained with Annexin V/PI and cell apoptosis was analyzed by flow cytometry (A) and expressed as mean ± standard deviation (B). Caspase-3, Caspase-8, Bax, and Bcl-2 proteins were detected by Western blotting (C, D). β-actin was used as the loading control. The data are shown as mean ± SD of three independent experiments each in triplicate. NS, not significant; **, p < 0.05; ***, p < 0.001.

To analyze the apoptosis of SH-SY5Y cells treated with Aβ, cells were stained with Annexin V/PI, and apoptotic cells were quantified by flow cytometry. As shown in Fig 3A and 3B, Aβ treatment significantly increased the apoptosis of SH-SY5Y cells, while overexpression of Tβ4 markedly decreased the Aβ-induced apoptosis.

Then, to examine the apoptosis-related signaling pathway, we collected total proteins from SH-SY5Y cells treated with 25 μM Aβ and examined the expression levels of cleaved Caspase-3, cleaved Caspase-8, Bax, and Bcl-2 by western blot. We found that the expression levels of proapoptotic Caspase-3, Caspase-8, and Bax were increased and the expression level of anti-apoptotic Bcl-2 was decreased after Aβ stimulus. Tβ4 overexpression reversed these effects, indicating that Tβ4 protected SH-SY5Y cells from apoptosis by regulating Bcl-2/Bax ratio and inhibiting Caspase-3 and Caspase-8 activation (Fig 3C and 3D).

### Tβ4 overexpression inhibits ERK/p38 MAPK pathway in Aβ-treated SH-SY5Y cells

**Fig 4:**
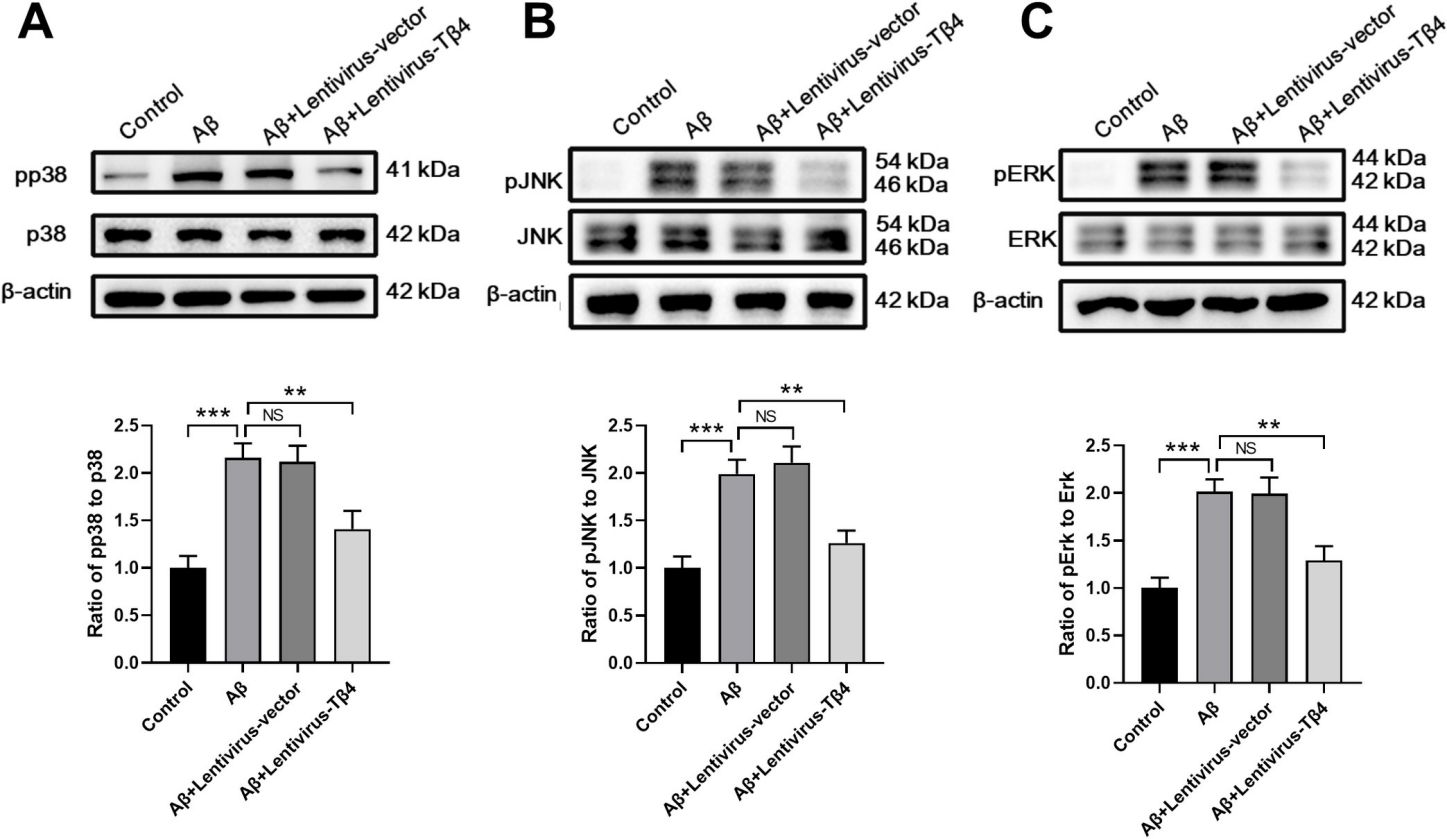
Effects of Tβ4 on ERK/p38 MAPK signaling in Aβ-treated SH-SY5Y cells. SH-SY5Y cells with or without overexpression of Tβ4 were treated with Aβ at a concentration of 25 μM for 48 h prior to western blot analysis to measure the levels of (A) p-p38/p38, (B) p-JNK/JNK and (C) p-ERK/ERK. β-actin was used as an internal control. The bars represent the mean ± SD in the different experimental groups (n = 3). NS, not significant; **, p < 0.01; ***, p < 0.001.

MAPK pathway was reported to be a key signaling pathway controlling cell survival [35]. To investigate the possible protective mechanism of Tβ4, we analyzed the three possible pathways downstream of MAPK, i.e. p38, JNK, and ERK. The results showed that the expressions of p-p38, p-JNK, p-ERK were significantly increased by Aβ stimulus while Tβ4 overexpression inhibited these increments, indicating that Tβ4 may protect SH-SY5Y cells from apoptosis by regulating the MAPK pathway (Fig 4).

### Tβ4 overexpression enhances 5-HTR1A expression in Aβ-treated SH-SY5Y cells

**Fig 5:**
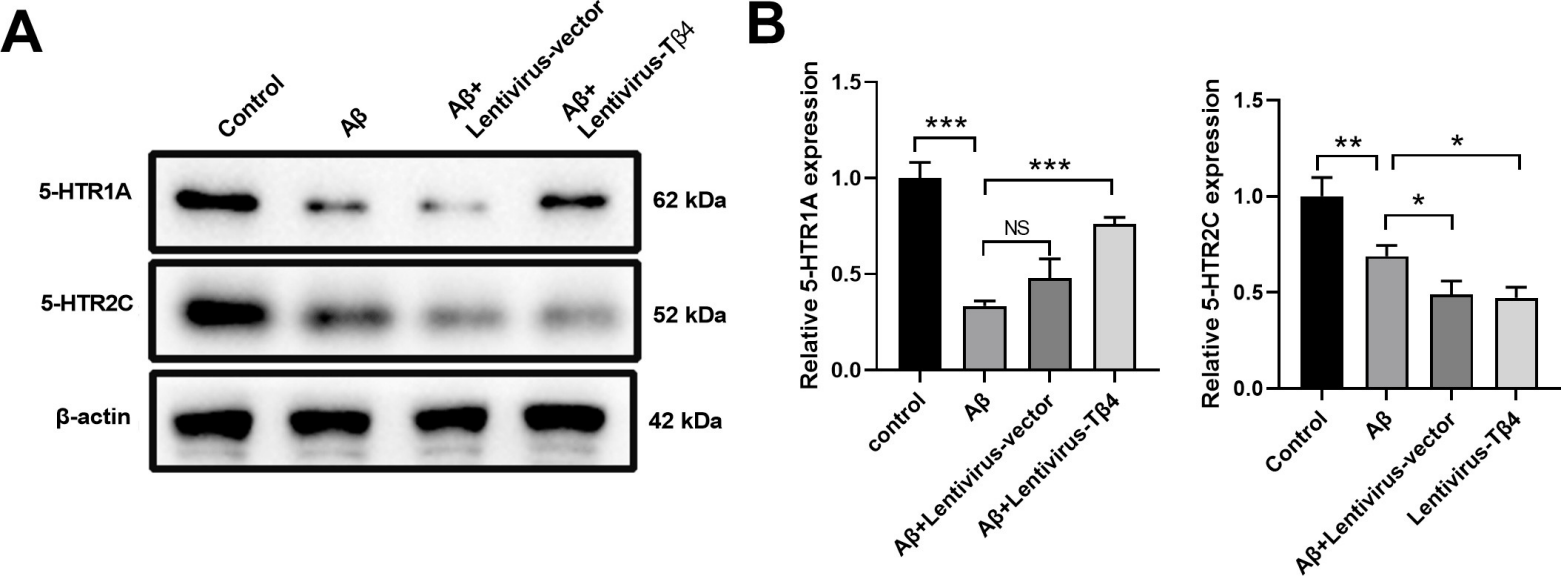
Effects of Tβ4 on 5-HTR1A and 5-HTR2C expression in Aβ-treated SH-SY5Y cells. SH-SY5Y cells with or without overexpression of Tβ4 were treated with Aβ at a concentration of 25 μM for 48 h prior to western blot analysis to measure the expression of 5-HTR1A and 5-HTR2C. β-actin was used as an internal control. The bars represent the mean ± SD in the different experimental groups (n = 3). NS, not significant; *, p < 0.05; **, p < 0.01; ***, p < 0.001.

Previous studies have shown that changes of 5-hydroxytryptamine receptors such as 5-HTR1A and 5-HTR2C were involved in the pathogenesis of AD [36]. Intriguingly, we found that compared with the control group, the relative expression of both 5-HTR1A and 5-HTR2C in the Aβ group decreased significantly (Fig 5). Overexpression of Tβ4 group enhanced the expression of 5- HTR1A but not 5-HTR2C expression, indicating that Tβ4 might have a greater impact on 5- HTR1A (Fig 5).

### Tβ4 inhibits ERK activation through up-regulating 5-HTR1A in Aβ-treated SH-SY5Y cells

**Fig 6:**
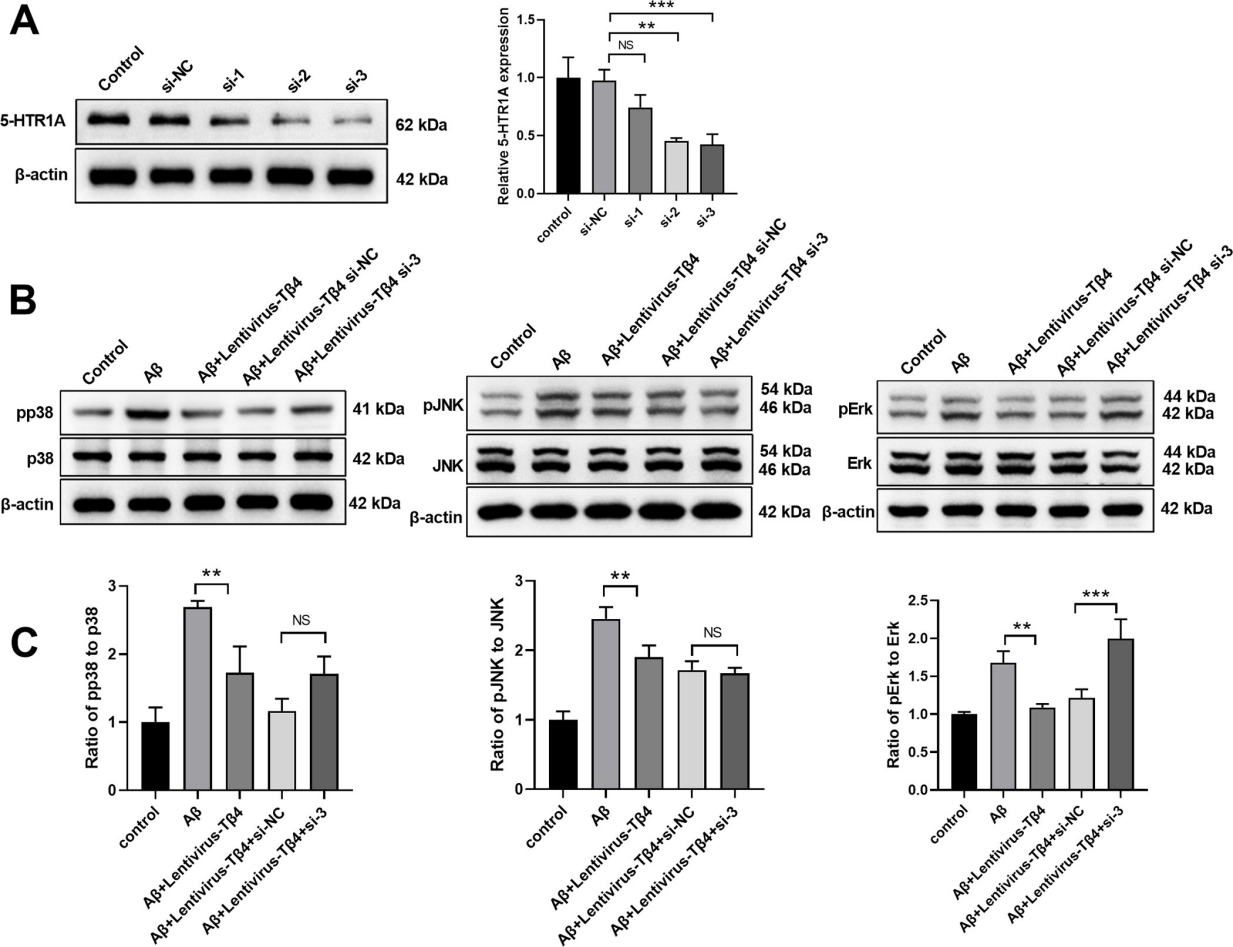
Tβ4 inhibits ERK activation through up-regulating 5-HTR1A in Aβ-treated SH-SY5Y cells. (A) SH-SY5Y cells were transfected with 5-HTR1A siRNAs for 48 h before the mRNA level of 5-HTR1A was quantified by qPCR. (B and C) Lentivirus-T4β transduced SH-SY5Y cells were transfected with 5-HTR1A siRNA and subsequently subjected to Aβ treatment at 25 μM for 48 h prior to western blot analysis to measure the expression of JNK/pJNK, p38/pp38 and ERK/pERK. β-actin was used as an internal control. The bars represent the mean ± SD in the different experimental groups (n = 3). NS, not significant; **, p < 0.01; ***, p < 0.001.

To examine the potential molecular signaling downstream of 5-HTR1A, we used siRNA to knockdown the expression of 5-HTR1A in SH-SY5Y cells. As shown in Fig 6A, compared with the si-NC group, 5-HTR1A siRNAs (si-2 and si-3) significantly silenced the expression of 5- HTR1A and si-3 was used for the subsequent experiments. When transfected with siRNA targeting 5-HTR1A in Tβ4-overexpressing cells, we found that the inhibitive effect of Tβ4 on ERK activation was diminished, indicating that Tβ4 might inhibit ERK activation through 5-HTR1A pathway (Fig 6B and 6C).

## Discussion

There is no effective treatment to prevent or cure AD and novel therapeutic strategies are urgently needed. Tβ4 is widely expressed in the Central Nervous System (CNS) and accumulating studies demonstrate that Tβ4 plays a key role in the protection and repair of CNS [37–39]. In the current study, we found that Tβ4 overexpression attenuates Aβ-induced cytotoxicity in neuroblastoma SH-SY5Y cells, showing enhanced cell viability and decreased cell apoptosis. In addition, Tβ4 overexpression suppressed oxidative levels and ROS production in Aβ-treated SH-SY5Y cells. We also demonstrated that Tβ4 enhanced 5-HTR1A expression while inhibited ERK/p38 MAPK signaling. In summary, our results suggest that Tβ4 could be a promising therapeutic target for the treatment of neurological diseases such as AD.

Conversion of Aβ from soluble monomer to aggregated form leads to the pathogenesis of AD [40]. Aβ-stimulated human neuroblastoma SH-SY5Y cells are widely used as an in vitro cellular model in biochemical and toxicological studies of AD [41]. We confirmed that Aβ treatment resulted in decreased cell viability and increased cell apoptosis in SH-SY5Y cells. These results are consistent with previous Aβ-indued neurotoxicity models [42–44]. However, Tβ4 overexpression using lentivirus transduction attenuated the cytotoxicity induced by Aβ. Mounting evidence demonstrates that oxidative stress is critical in the initiation and progression of AD [45]. In this study, it was observed that Aβ treatment led to an increase in MDA and LDH levels, while causing a decrease in SOD and GSH-PX activities. Interestingly, when Tβ4 was overexpressed, it significantly diminished oxidative stress by enhancing SOD and GSH-PX activities, and reducing LDH activity and MDA level in Aβ-treated SH-SY5Y cells. Consistently, we found that Tβ4 overexpression suppressed ROS production in Aβ-stimulated SH-SY5Y cells.

Oxidative stress, ROS, and Aβ could cause neuronal death via apoptosis in AD [46]. We found that overexpression of Tβ4 inhibited Aβ-induced apoptosis in SH-SY5Y cells. The apoptotic signaling cascade was analyzed and the results showed that Aβ treatment enhanced the expression of pro-apoptotic Caspase-3, Caspase-8, and Bax while suppressed the expression level of anti-apoptotic Bcl-2. Tβ4 overexpression abolished these effects, indicating that Tβ4 protected SHSY5Y cells from apoptosis by regulating Bcl-2/Bax ratio and inhibiting Caspase-3 and Caspase-8 activation. Previous studies have shown that Tβ4 is a secretive protein that functions in both autocrine and paracrine manners [47,48]. In this study we used lentiviral vectors to upregulate the endogenous expression of Tβ4 in SH-SY5Y cells and demonstrated the neuroprotective function of Tβ4. One of the limitations of the current study is that whether the ERK/MAPK and 5-HT1A Signaling Pathway, which regulated by endogenous Tβ4 is applicable to secretive Tβ4 is unknown. Another limitation of this study is that we used Aβ-induced apoptosis as an in vitro neurodegenerative model. Whether Tβ4 has neuroprotective function to other neurodegenerative models such as α-synuclein-induced Parkinson disease model [49], hypoxia induced neurodegeneration [50], and N10-substituted phenoxazine induced Huntington disease model [51].

MAPK signaling pathway plays important role in the development of AD [52]. Previous studies have demonstrated that ROS activates JNK and MAPK signaling pathways in AD [53]. ERK1/2 signaling was reported to be activated by ROS and promote cell apoptosis [54]. Changes of 5- hydroxytryptamine receptors such as 5-HTR1A and 5-HTR2C were involved in the pathogenesis of AD [36]. However, the regulation between 5-hydroxytryptamine receptors and signaling pathways are not clear. We found that expressions of p-p38, p-JNK, p-ERK were significantly increased by Aβ stimulus while Tβ4 overexpression inhibited these increments, indicating that Tβ4 may protect SH-SY5Y cells from apoptosis by regulating the MAPK pathway. Intriguingly, overexpression of Tβ4 group only enhanced the expression of 5-HTR1A but not 5-HTR2C expression. Moreover, we demonstrated that Tβ4 overexpression inhibited the phosphorylation of p38 and JNK, but could not inhibit ERK activation in SH-SY5Y cells after knocking down 5- HTR1A, indicating that Tβ4 might inhibit ERK activation through the 5-HTR1A pathway. Therefore, our data suggest that the Tβ4/5-HTR1A/ERK signaling axis plays an essential role in the pathogenesis AD.

Taken together, our findings provide evidences to support the neuroprotective role of Tβ4 and might open up new therapeutic applications of Tβ4 in AD treatment. Further detailed investigations are needed to fully understand the mechanisms of action of Tβ4 in AD. The function of Tβ4 on astrocyte/oligodendrocyte/neural stem cell differentiation is not depicted in the current study. Whether Tβ4 plays a similar protective role in the animal AD model should also be addressed.

## Supporting information

S1 Raw images (PDF)

S1 Dataset (RAR)
