# Supplementary material for: Antioxioxidant and antiapoptotic effects of Thymosin β4 in Aβ-induced SH-SY5Y cells via the 5-HTR1A/ERK axis
Source: PLoS One. 2023 Oct 3;18(10):e0287817. doi: 10.1371/journal.pone.0287817 (PMC10547165; doi:10.1371/journal.pone.0287817)
Supplement: S1 Raw images — (PDF) [file pone.0287817.s001.pdf]

Fig 1C:

(1) The loading order: Control group, Lentivirus empty vector group and Lentivirus-Tβ4 group.

(2) Identity of experimental samples: cells.

(3) Method used to capture the image: Chemiluminescence

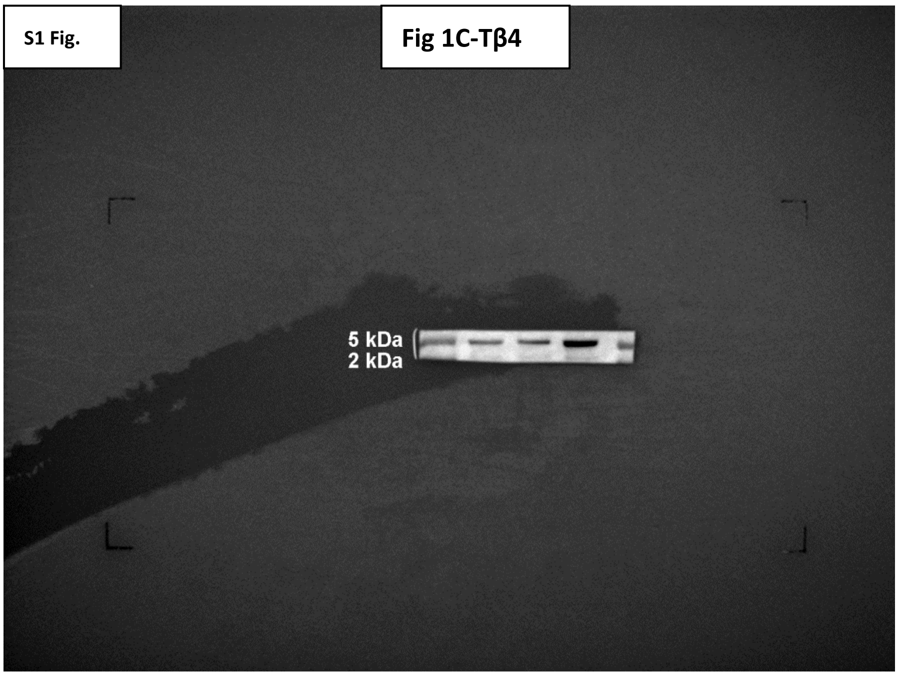

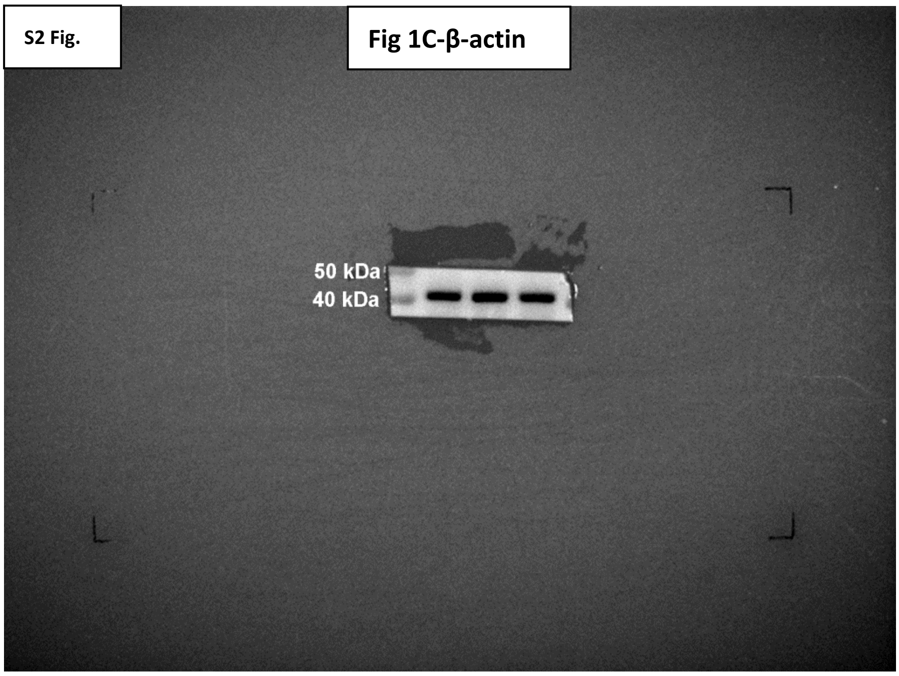

Fig 3C:

(1) The loading order: Control group, Aβ group, Aβ+Lentivirus empty vector group and Aβ+Lentivirus-Tβ4 group.

(2) Identity of experimental samples: cells.

(3) Method used to capture the image: Chemiluminescence

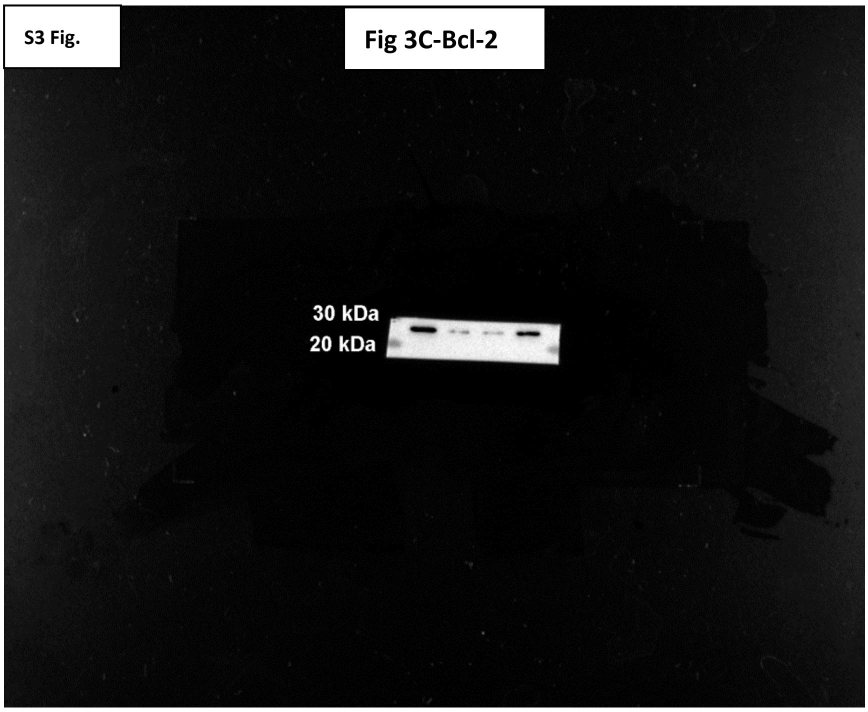

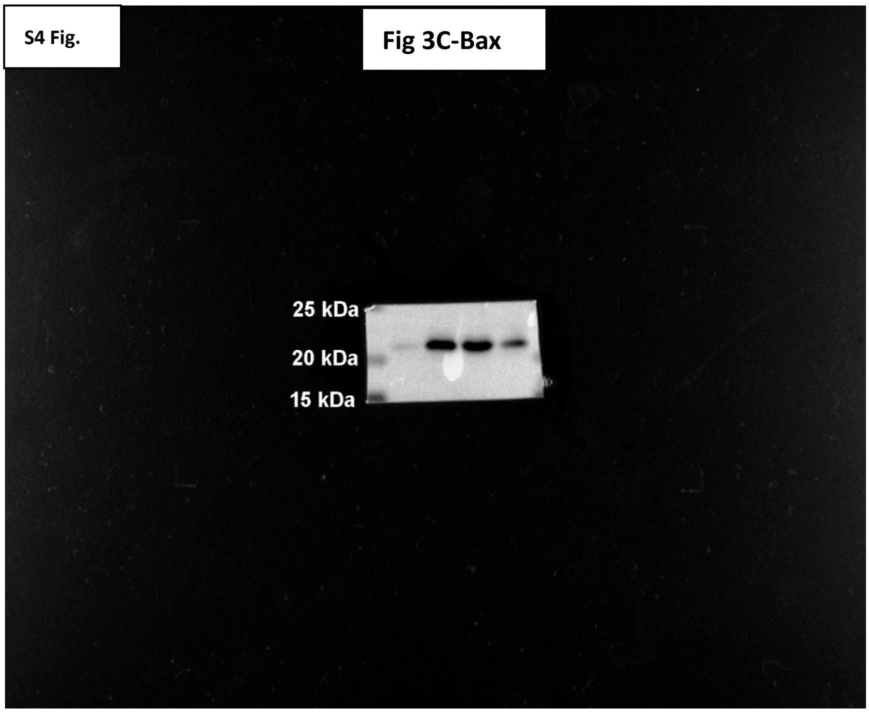

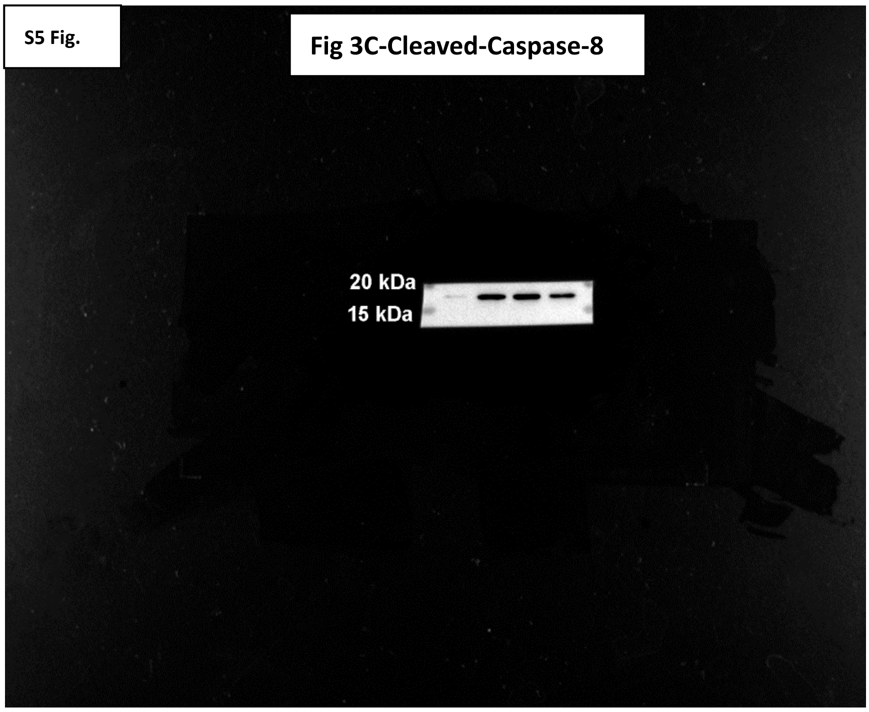

S5 Fig.

Fig 3C-Cleaved-Caspase-8

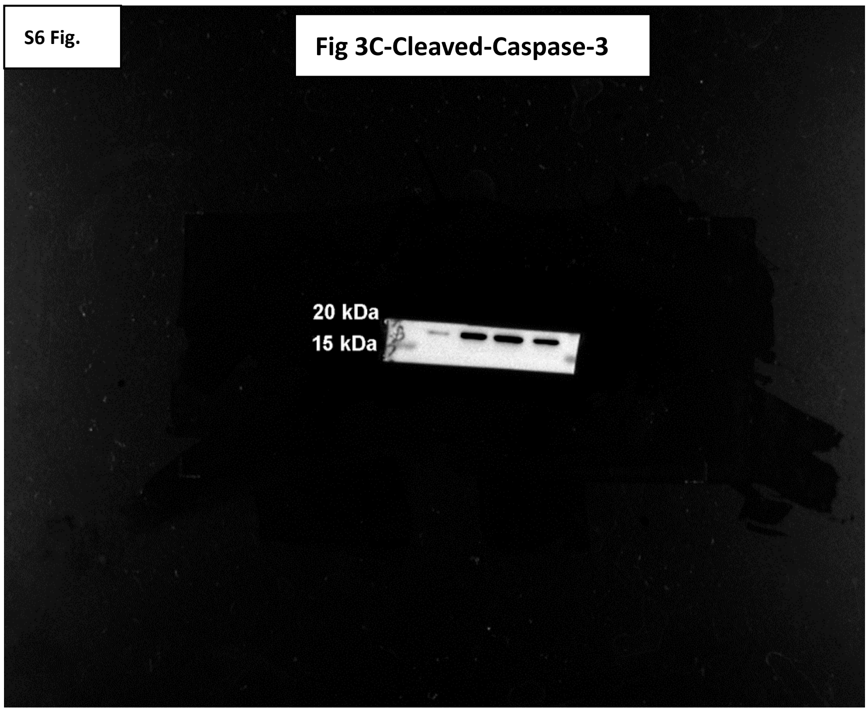

S6 Fig.

Fig 3C-Cleaved-Caspase-3

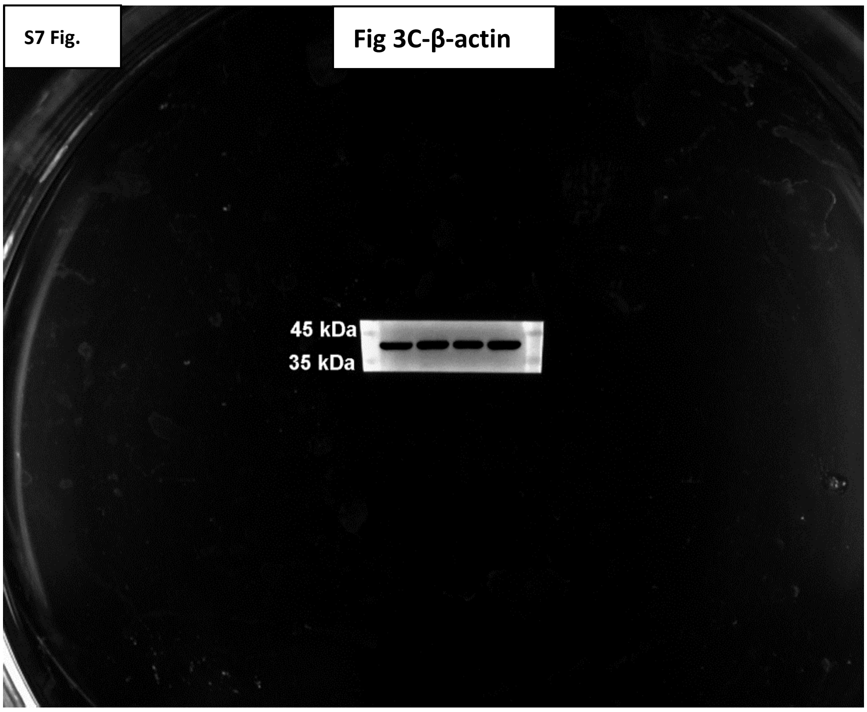

Fig 4A:

(1) The loading order: Control group, Aβ group, Aβ+Lentivirus empty vector group and Aβ+Lentivirus-Tβ4 group.

(2) Identity of experimental samples: cells.

(3) Method used to capture the image: Chemiluminescence

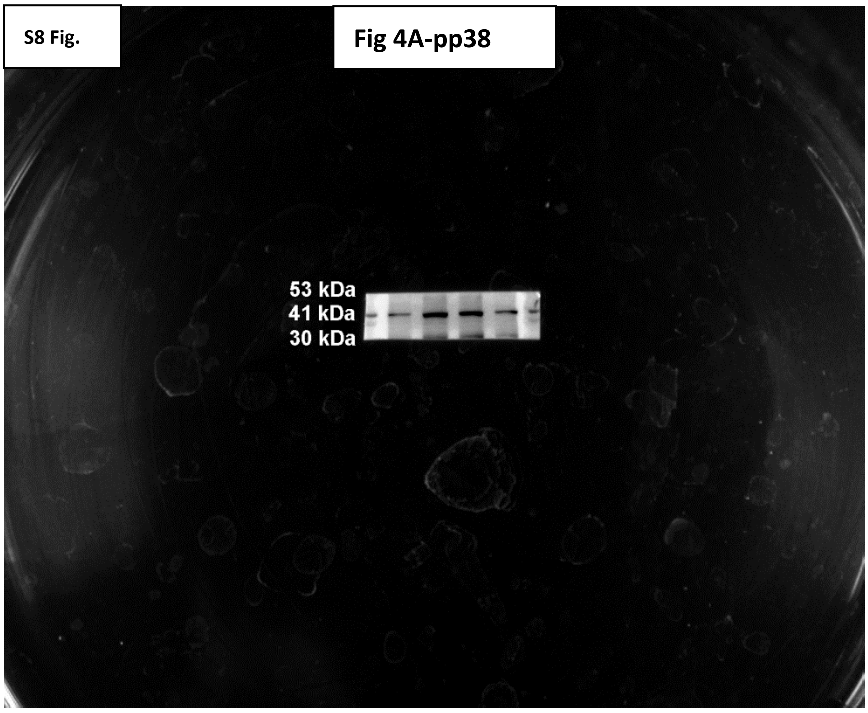

S9 Fig.

Fig 4A-p38

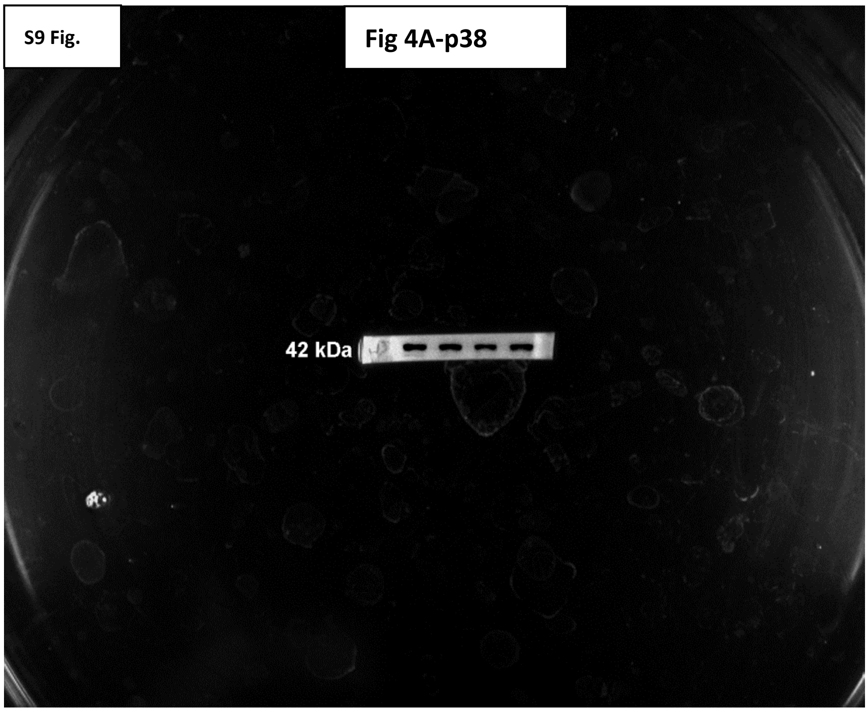

S10 Fig.

Fig 4A-β-actin (p38)

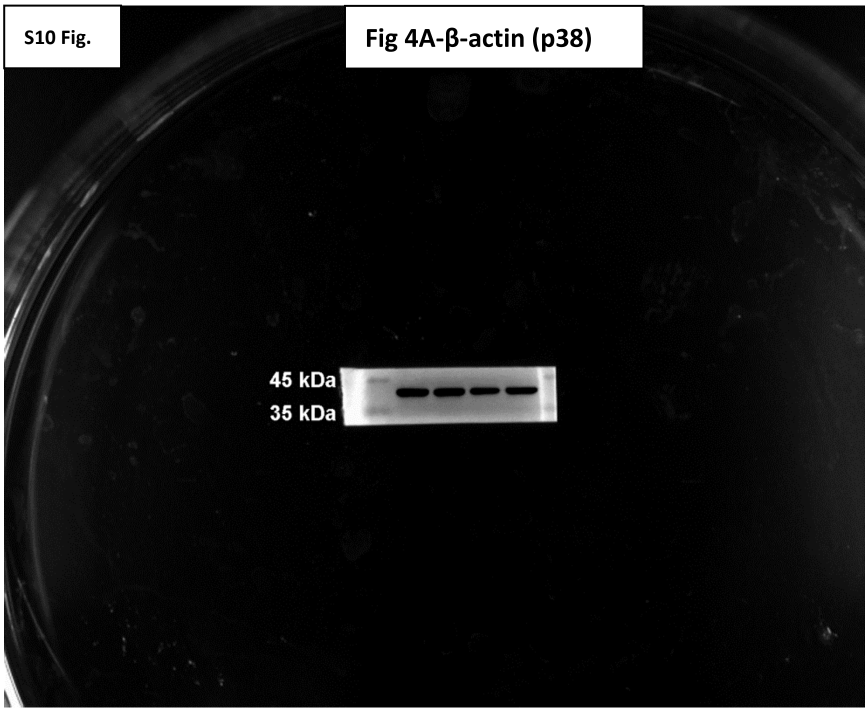

S11 Fig.

Fig 4A-pJNK

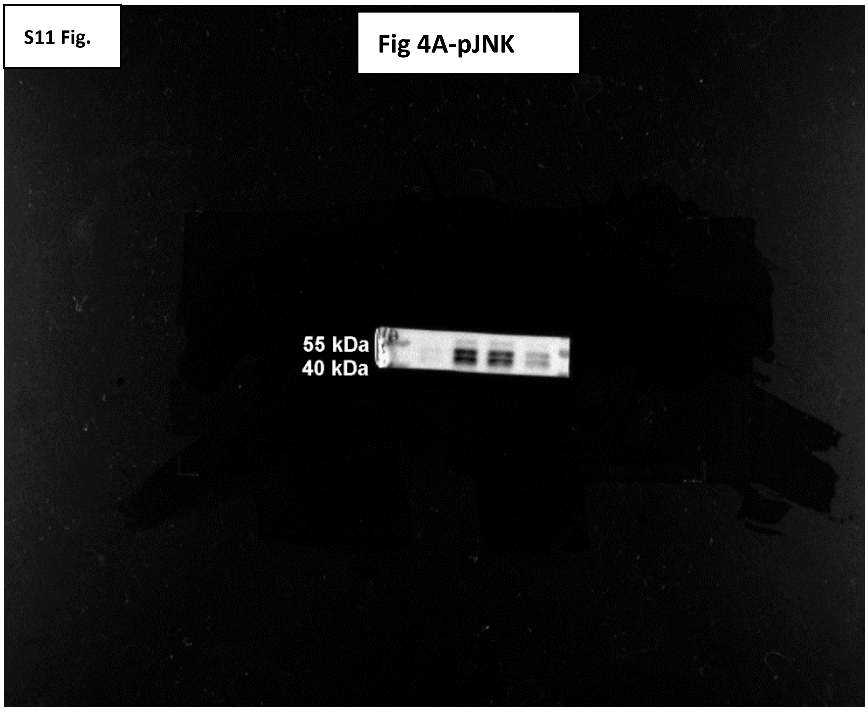

S12 Fig.

Fig 4A-JNK

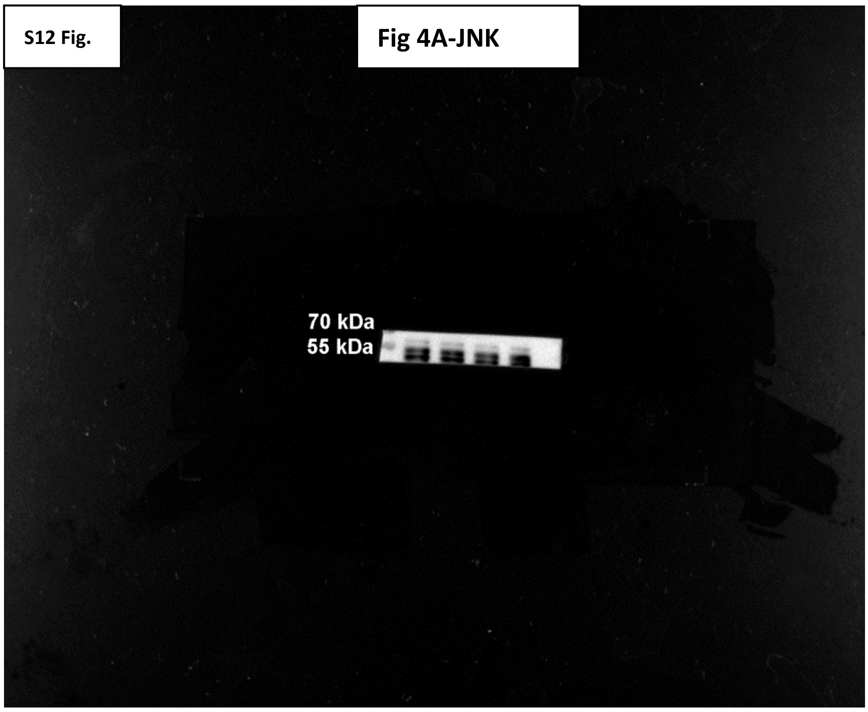

S13 Fig.

Fig 4A-β-actin (JNK)

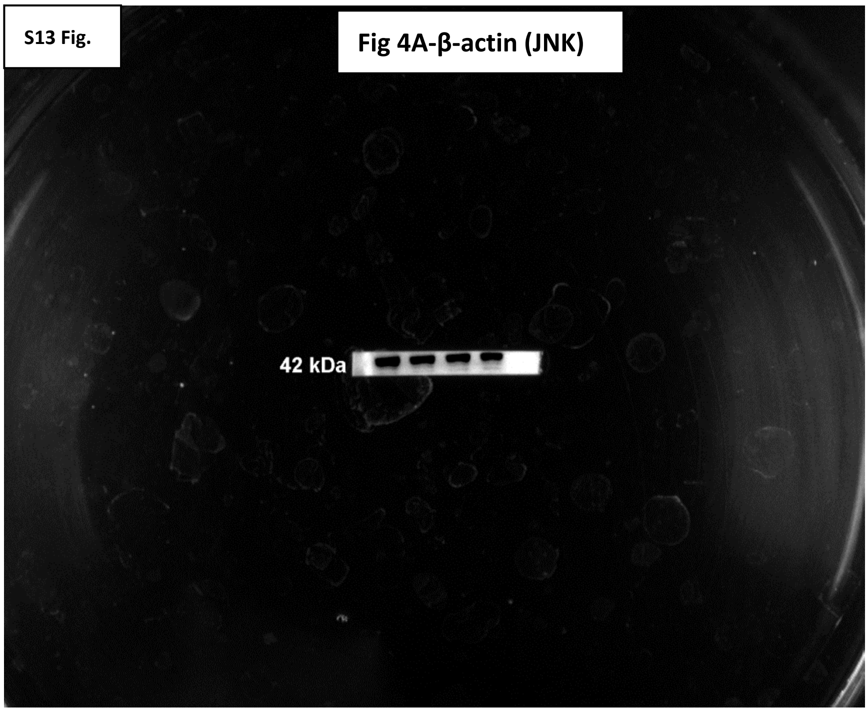

S14 Fig.

Fig 4A-pERK

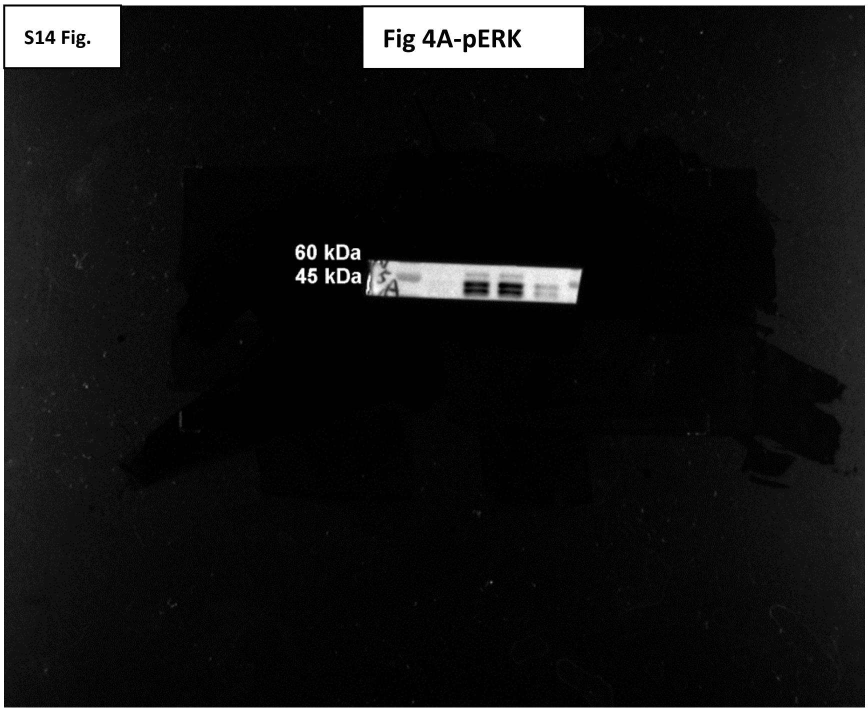

S15 Fig.

Fig 4A-ERK

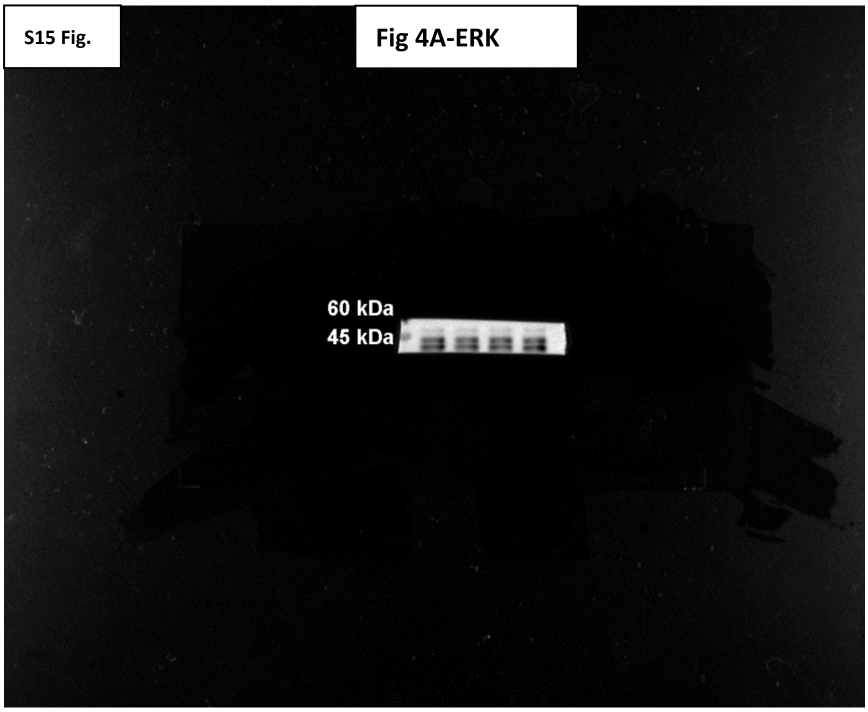

S16 Fig.

Fig 4A-β-actin (ERK)

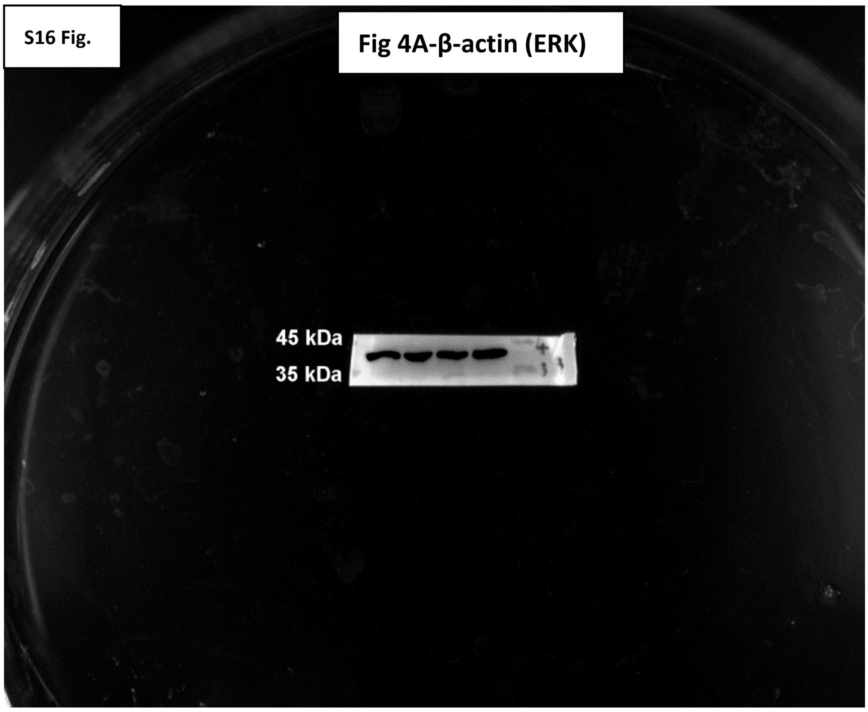

Fig 5A:

(1) The loading order: Control group, Aβ group, Aβ+Lentivirus empty vector group and Aβ+Lentivirus-Tβ4 group.

(2) Identity of experimental samples: cells.

(3) Method used to capture the image: Chemiluminescence

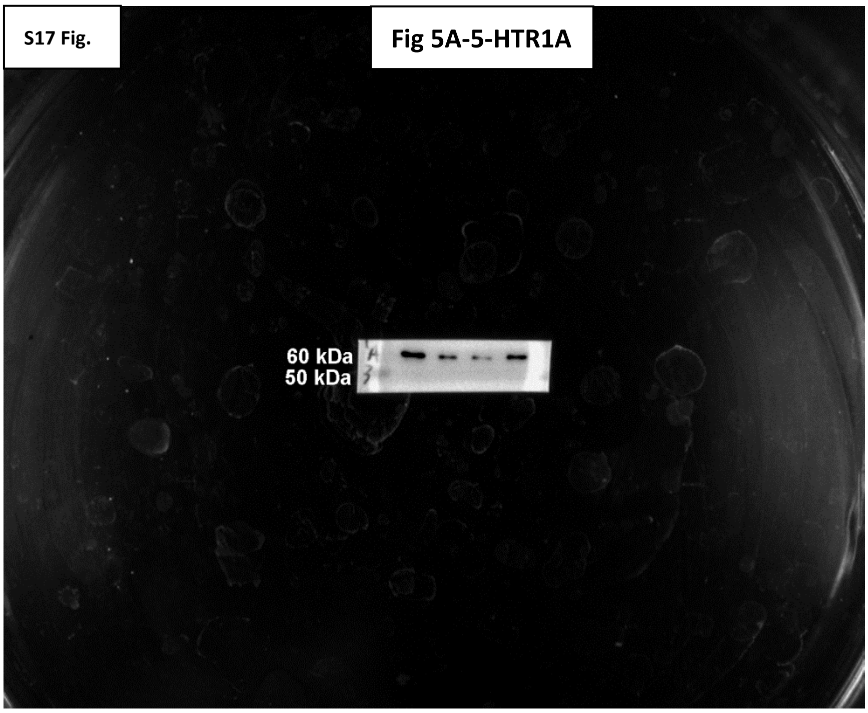

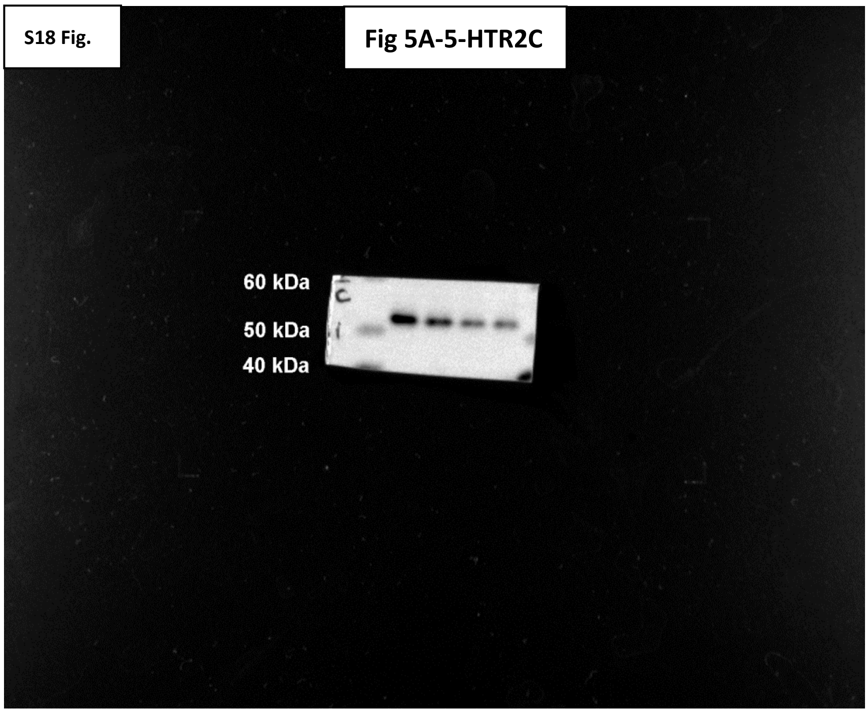

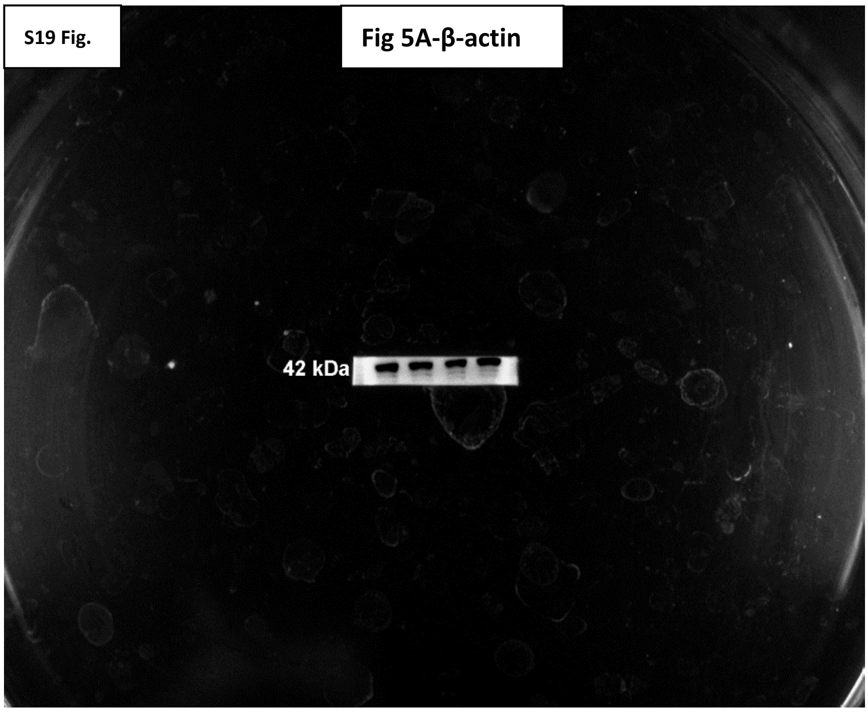

Fig 6A:

(1) The loading order: Control group, si-NC group, si-1 group, si-2 group and si-3 group.

(2) Identity of experimental samples: cells.

(3) Method used to capture the image: Chemiluminescence

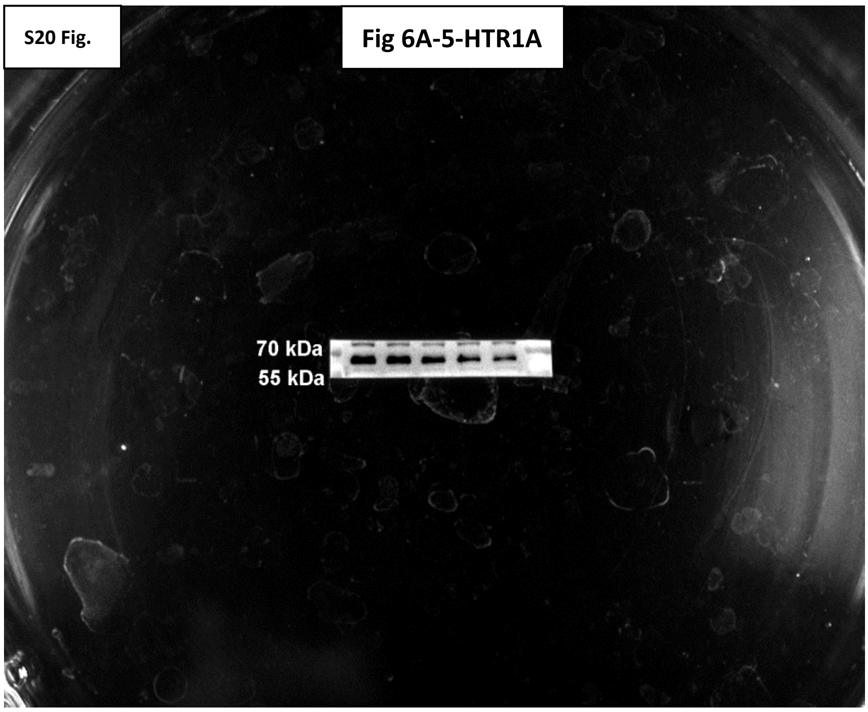

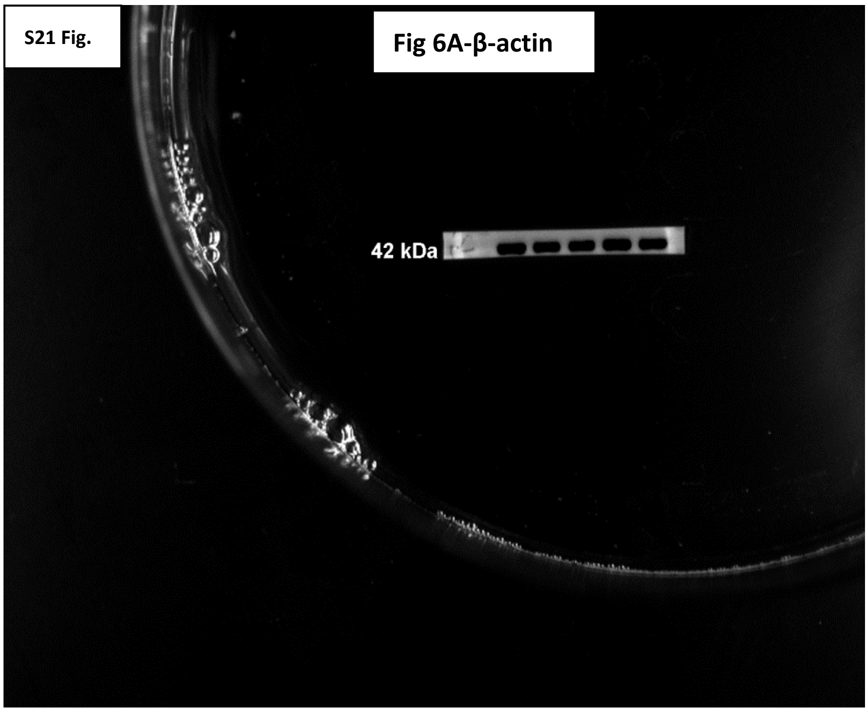

Fig 6B:

(1) The loading order: Control group, Aβ group, Aβ+Lentivirus-Tβ4, Aβ+Lentivirus-Tβ4 si-NC group and Aβ+Lentivirus-Tβ4 si-3 group.

(2) Identity of experimental samples: cells.

(3) Method used to capture the image: Chemiluminescence

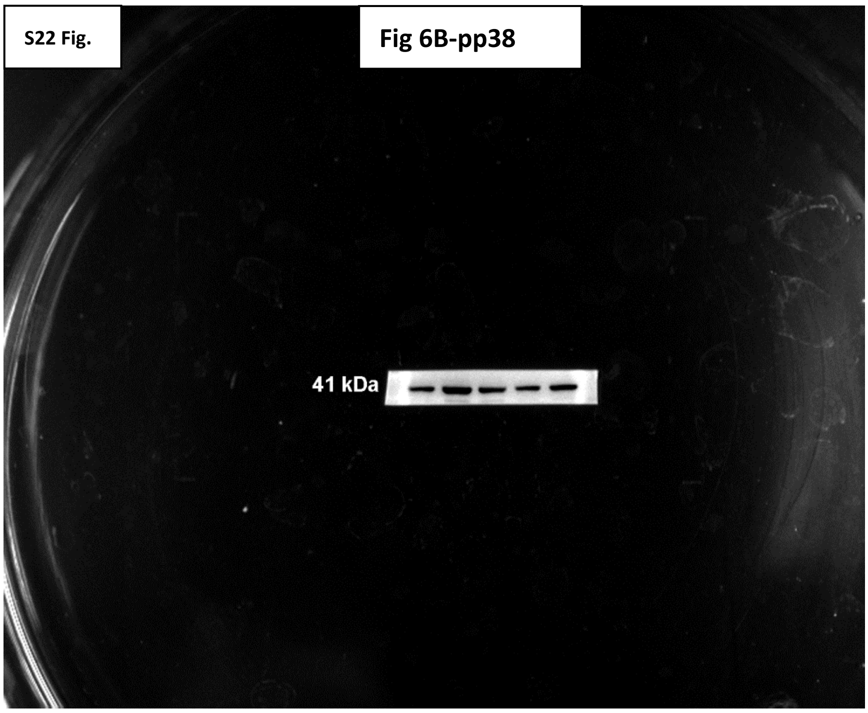

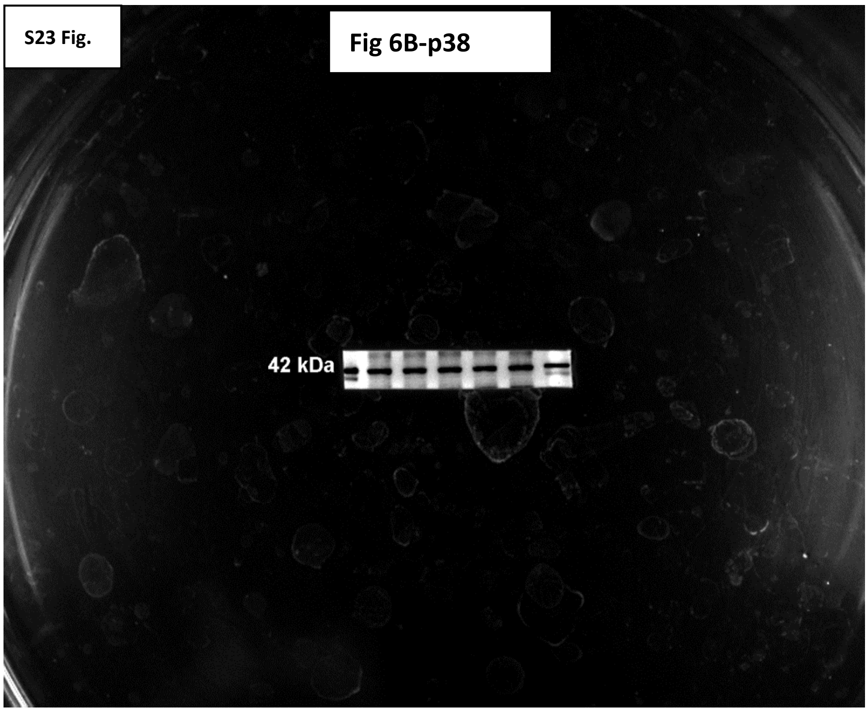
S23 Fig.
Fig 6B-p38
42 kDa

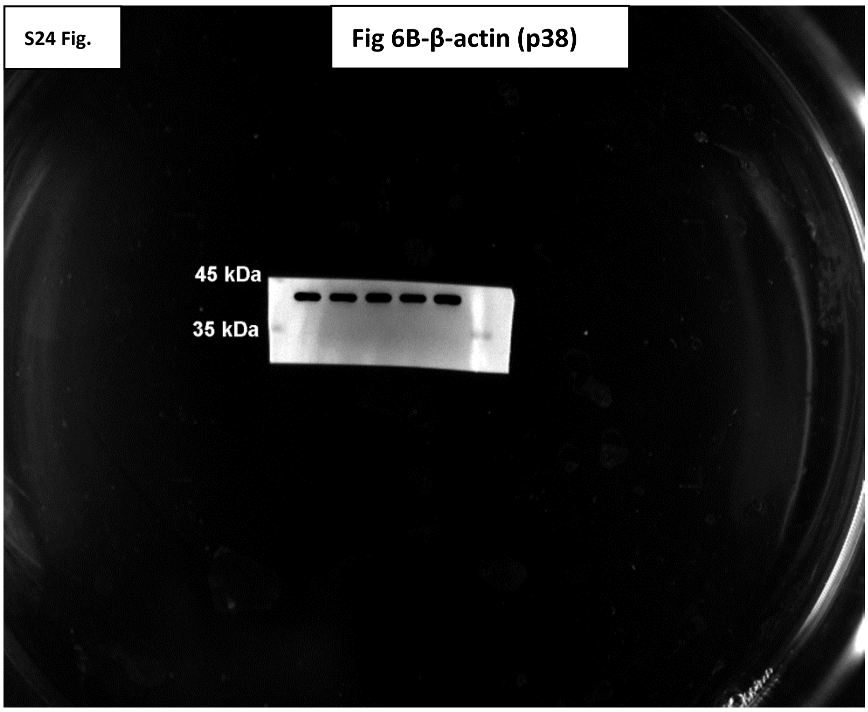
S24 Fig.
Fig 6B-β-actin (p38)
45 kDa
35 kDa

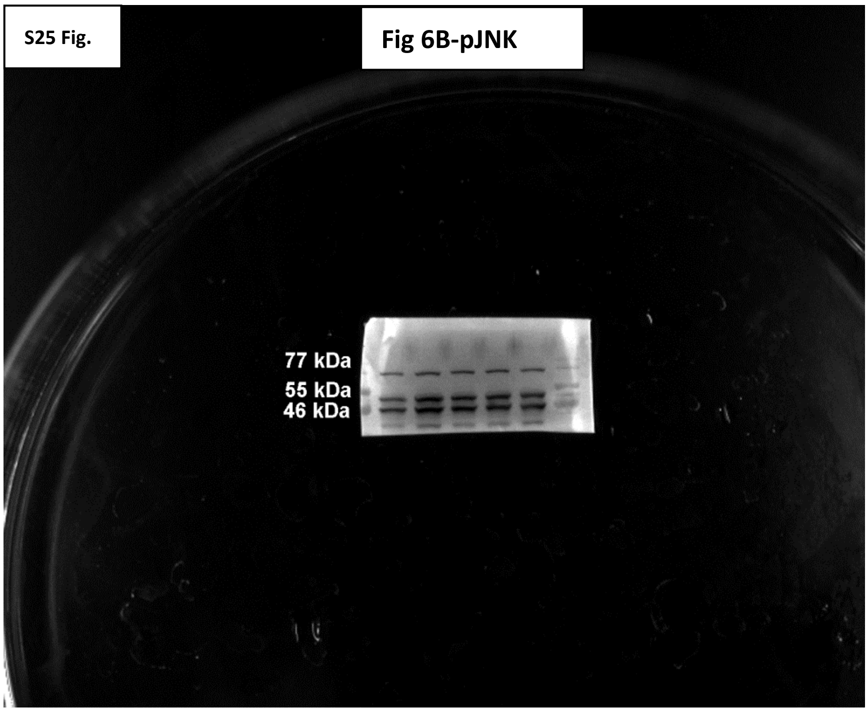
S25 Fig.
Fig 6B-pJNK
77 kDa
55 kDa
46 kDa

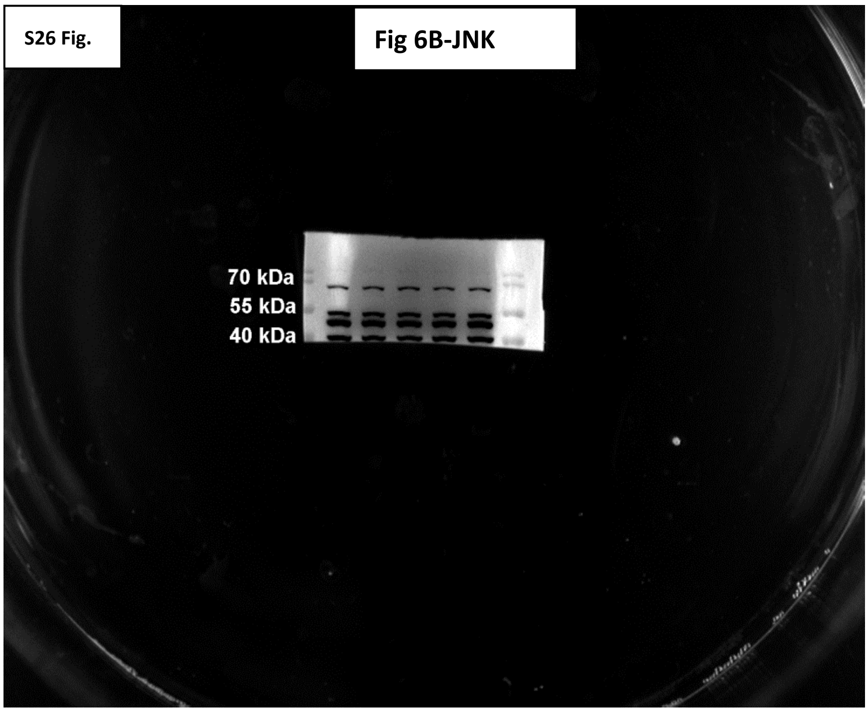
S26 Fig.
Fig 6B-JNK
70 kDa
55 kDa
40 kDa

S27 Fig.

Fig 6B-β-actin (JNK)

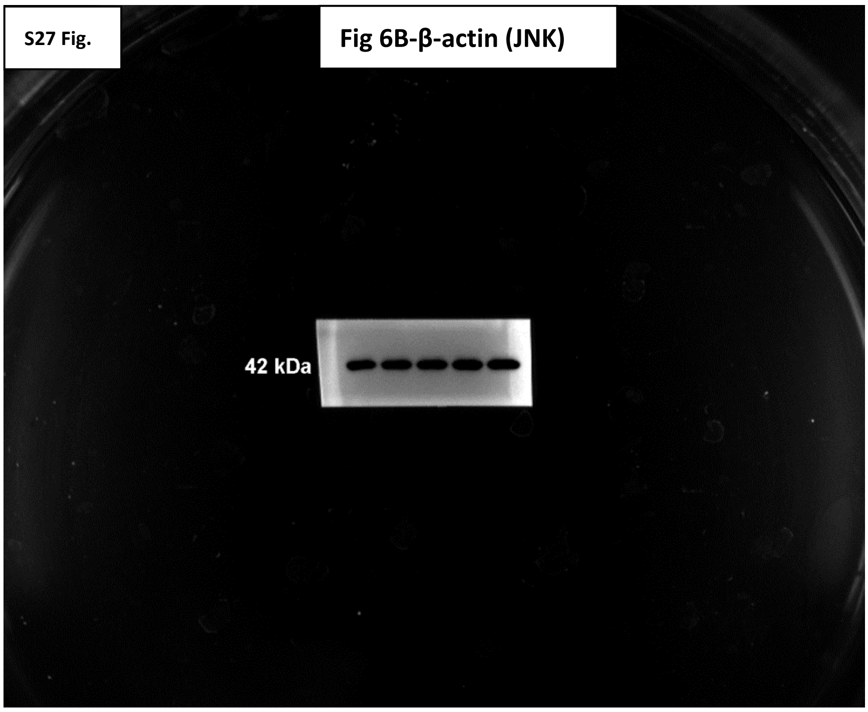

S28 Fig.

Fig 6B-pERK

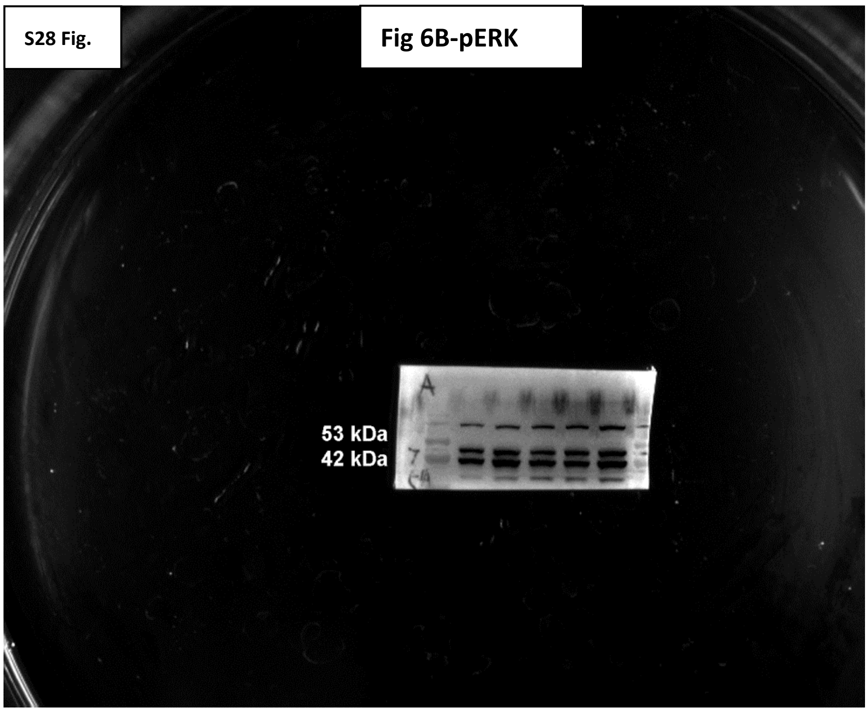

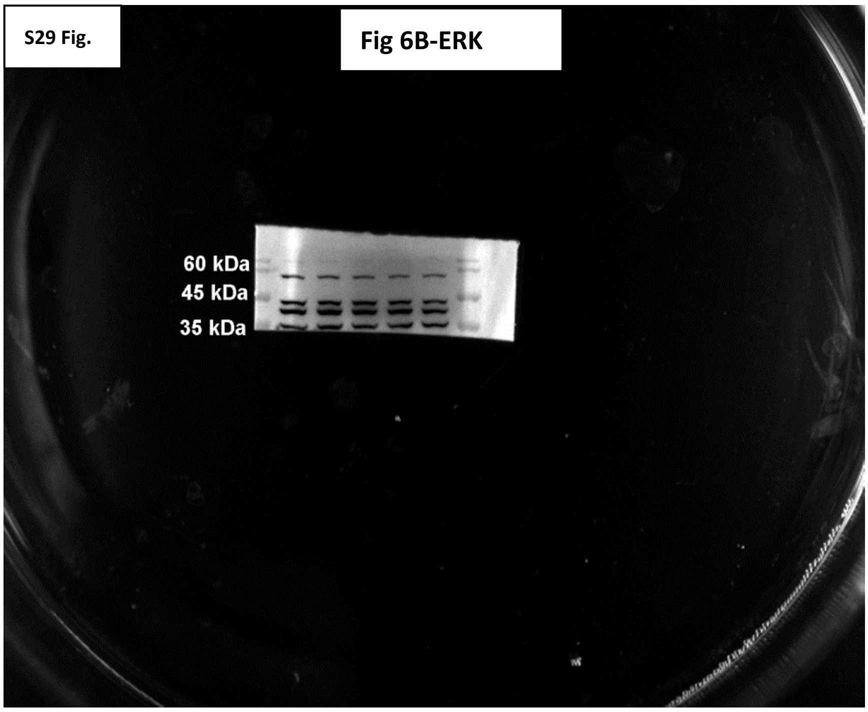
S29 Fig.
Fig 6B-ERK
60 kDa
45 kDa
35 kDa

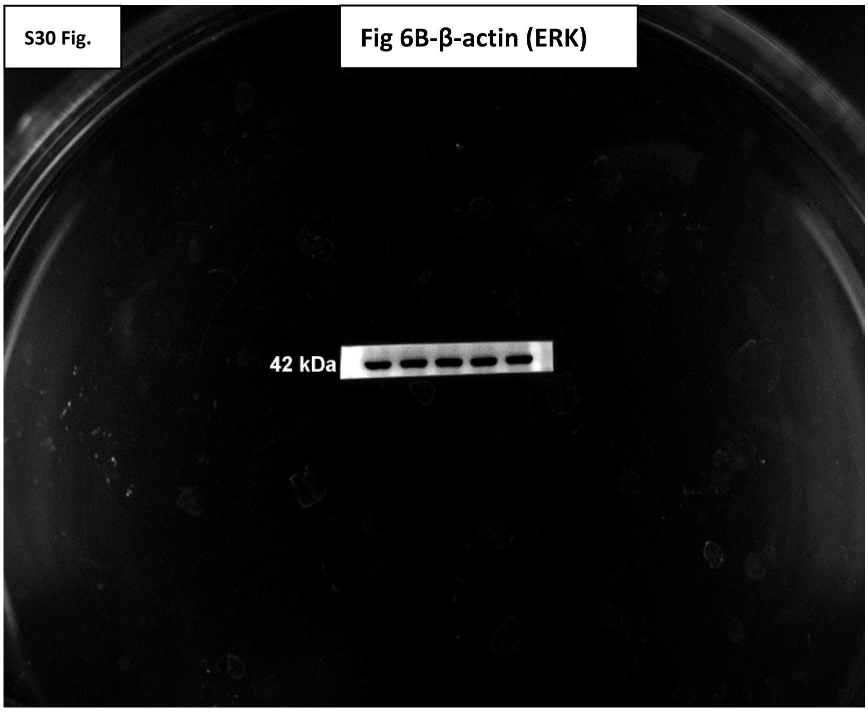
S30 Fig.
Fig 6B-β-actin (ERK)
42 kDa
